# A new *Pseudachorutes* (Collembola, Neanuridae, Pseudachorutinae) from Nicaragua

**DOI:** 10.3897/zookeys.187.2362

**Published:** 2012-04-27

**Authors:** José G. Palacios-Vargas, Hugo H. Mejía-Madrid

**Affiliations:** 1 Laboratorio de Ecología y Sistemática de Microartrópodos, Depto. Ecología y Recursos Naturales, Facultad de Ciencias, UNAM, Circuito Exterior s/n, Ciudad Universitaria C.P. 04510, México D. F.

**Keywords:** Neanuridae, Taxonomy, Nicaragua

## Abstract

A new species of *Pseudachorutes* is described and illustrated from Nicaragua. *Pseudachorutes nica* **sp. n.** is very easy to distinguish from other members of this genus from Central America, because its chaetotaxy consists of macro and microsetae and a postantennal organ with multiple vesicles (close to 20).

## Introduction

*Pseudachorutes* is a very large genus with more than 100 species described all over the world. It was established by Tullberg (1871) based on the type species *Pseudachorutes subcrassus*. The genus is characterized by: 1) ocelli 8+8; 2) postantennal organ in one circle or one ellipse; 3) Ant. III and IV dorsally fused, Ant. IV generally with 6 sensilla and apical bulb, Ant. III organ with 2 microsensilla in a cuticular fold, 2 guard sensilla and one microsensillum; 4) buccal cone sharp, mandible with 2 or more teeth, maxilla styliform; 5) unguiculus absent; 6) furcula usually well developed, mucro present (except *Pseudachorutes amucronatus* Díaz & Najt, 1995); 7) sixth abdominal segment always visible in dorsal view, anal spines absent (Palacios-Vargas 1990; Christiansen and Bellinger 1998; Fjellberg 1998).

Only *Pseudachorutes orghidani* Massoud and Gruia, 1969, from Cuba, *Pseudachorutes difficilis* Denis, 1931, from Costa Rica, *Pseudachorutes legrisi* Thibaud and Massoud, 1983, and *Pseudachorutes reductus* Thibaud and Massoud, 1983 both from the Antilles, have been described from Central America and neighboring areas. Additional records include *Pseudachorutes* sp. and *Pseudachorutes subcrassoides* from Nicaragua (Maes and Palacios-Vargas 1988), and *Pseudachorutes parvulus* from Cuba, a species originally described from Europe (Díaz-Azpiazu et al. 1996).

While studying the Collembola material from a project of the Centre de Recerca Ecologica i Aplicacions Forestals of Barcelona (CREAF) in Nicaragua, we found many specimens of a new species which is described herein.

Abbreviations used in this paper are: Ant. = antennal segment; Abd. = abdominal tergite; M = macroseta; m = microseta; PAO = postantennal organ; vgs = ventral guard sensillum; Th. = thoraxic tergite. Chaetotaxy follows Jordana et al. (1997).

## Taxonomy

### Pseudachorutes nica sp. n.

urn:lsid:zoobank.org:act:61AA57D8-D9F5-4AF7-8137-59225D1871F6

http://species-id.net/wiki/Pseudachorutes_nica

Figs 1 –11

#### Material Examined.

**Type**-**locality:** Nicaragua, Province Estelí: Mesas de Moropotente, 12°55'09"N; 85°11'90"W; ex soil, *Acacia pennatula*, *Oplinemus*, *Croton jalapensis*, forest, 23 August 2007, ex soil from pitfall traps, Pilar Andrés collector.

#### Type-specimen.

Holotype mounted on slide. Original label: “23/8/07 Nicaragua: Mesas de Moropotente (Estelí). 1260 m.s.n.m. Coord. UTM 16P0581619 y 1456016 Suelo Bosque monoespecífico *Acacia pennatula* Recub. *Oplisnemus* sp. (100%) P. Andrés, col. Sample C3 (8)” [printed label] “ *Pseudachorutes nica* sp. n. Holotipo ♀ C3 (8)” [handwritten label].

#### Paratypes.

55 females, 38 males, 9 juveniles, 33 of undetermined sex, all under slides. Two females and two males will be deposited in Museum National d’Histoire Naturelle de Paris (MNHNP) and Museum d’Histoire Naturelle de Genève (MHNG), the others are kept at Facultad de Ciencias, UNAM.

#### Diagnosis.

Postantennal organ with about 20 vesicles in an ellipse; ocular area, head, and body with setae of different size; Ant. IV with a well developed ventral sensorial file with 20–25 setae; one lateral teeth on each side of unguis, presence of setae d1 unpaired on head and m2 on Th. II. Abd. IV with three rows of setae, p1 longer than seta p2 and a1. No capitate tenent hairs on tibiotarsi.

#### Description.

Body length: 1.1–1.6 mm (mean 1.4 mm, n=10). Color of body deep dark blue. Cuticular granulation strong. Body setae simple and smooth, medium macrosetae (25–35 µm) withslightly capitate tips, short microsetae (9–12 µm) usually acuminate, sensorial setae longer than macrosetae (60 – 81 µm)**,** with blunt apex.

**Figures 1–6.**
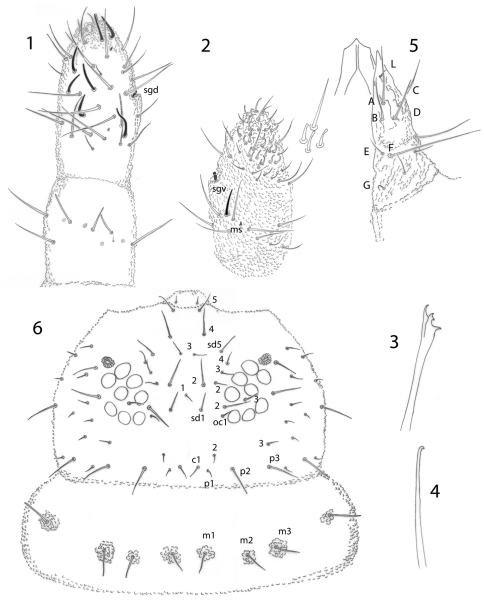
*Pseudachorutes nica* sp. n. **1** right antenna from II to IV dorsal view **2** Ant. III and IV in ventral view with magnification of some setae from ventral file **3** mandible **4** maxilla **5** labium **6** dorsal chaetotaxy of the head and thorax I (thorax has a drawing style represents granulation close to setae).

Antennae as long as head. Ant. I with 7 setae, Ant. II with 12 setae, including one ventral seta very small. Ant. III and IV dorsally fused. Antennal segments ratio I: II: III+IV as 1: 1.25–2.75. Sensory organ of Ant. III with two small straight internal sensilla under a cuticular fold, two guard sensilla, and 1 microsensillum close to vgs. Ant. IV with trilobed apical bulb, 6 cylindrical sensilla, seta “i”, microsensillum and subapical organite (Fig. 1), and with a ventral file with about 20–25 short setae (Fig. 2).

**Figures 7–10.**
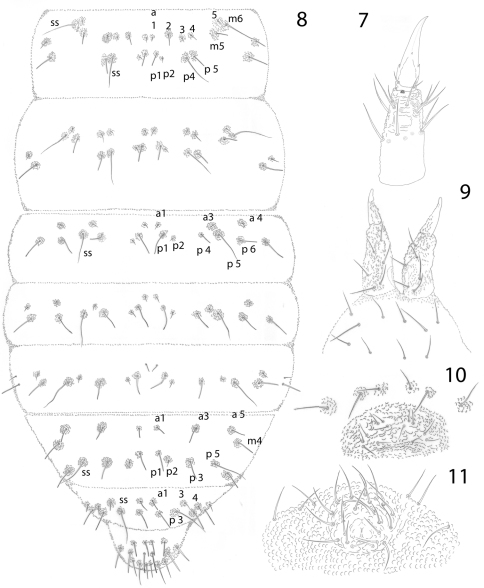
*Pseudachorutes nica* sp. n. **7** tibiotarsus III and unguis in ventral view **8** dorsal chaetotaxy of Th. II and III and Abd. I–VI (drawing style represents granulation close to setae) **9** furcula **10** female genital plate **11** male genital plate.

Postantennal organ elliptical composed of 14–20 simple vesicles, as large as the nearby ocelli. Eye patch with 8+8 small ocelli (Fig. 6), F, G a little smaller than others. Buccal cone elongated. Mandible with four slender teeth (Fig. 3). Maxilla styliform, with one apical hook (Fig. 4). Labrum with 2/5, 5, 2 setae, the sclerotization in the shape of ogive (Fig. 5). Labium with typical number ofsetae for the genus and seta L situated on small tubercle (Fig. 5).

Dorsal chaetotaxy as in Figs 6 and 8. Seta a0 on the head absent, unpaired seta d1 present. Th. I with 3+3 dorsal setae. Setae a2 present on Th. II, but absent from Th. III to Abd. IV, with m4 present on Abd. IV. Sensory setae on the body in position of p4 and m6 on Th. II and III, and p5 from Abd. I–IV and p3 on Abd. V. Sensorial formula of the body 022/11111. Sensory setae longer and slender than ordinary setae. Ratio of unguis III: largest Abd. V seta = 1: 0.5. Thoracic sterna without setae, but paratergal areas of Th. II and III with two setae on each side. Ventral tube with 4+4 setae, the posterior pair is longer than the others. Female genital plate with 3+3 pregenital, 7–12 circumgenital and 2 eugenital setae (Fig. 10); male genital plate with 3+3 pregenital, 10–13 circumgenital and 4+4 to 6+6 eugenital setae (Fig. 11). Each anal valve with 13 setae and 3 hr setae.

Tibiotarsi I, II, III with 18, 18, 17, setae respectively, without tenent hairs. Unguis with one tooth on each side, 1/3 from base, and a tiny inconspicuous tooth on inner edge. Ratio tibiotarsus III: unguis about 1.3 (Fig. 7). Femora I, II, III with 11, 11, 10 setae respectively, one of them longer and acuminate. Trochanters with 5 setae each.

Furcula well developed. Manubrium with one pair of dorsal longer setae. Dens with moderate granulation dorsally and with 6 setae, with a smooth triangular area ventrally devoid of secondary granulation. Mucro about half the length of dens, triangular, with two very short lamellae and granulation similar to dens, apex slightly curved (Fig. 9). Tenaculum with 3+3 teeth.

Etymology**.** The name is derived from the country nickname that is the type locality.

#### Distribution.

Known only from type locality. Province Estelí: Mesas de Moropotente, *Acacia pennatula*, *Oplinemus*, *Croton jalapensis* forest, 23 August 2007, ex soil from pit fall traps.

#### Ecology.

More than 135 specimens were found in the samples of pitfall traps from two different pasture fields on August 23, 2007.The species is very abundant in most of the collecting traps.

#### Discussion.

*Pseudachorutes nica* sp. n. is easily distinguished from all other species of the genus by the combination of the following characters: presence of many vesicles in the postantennal organ, well-differentiated macrosetae and microsetae on dorsal side of head and body, one ventral sensorial file on Ant. IV, presence of one lateral tooth on each side of unguis, presence of setae d1 unpaired on the head and presence of setae m2 on Th. II. On Abd. IV p1 is longer than p2 and a1. The new species is close to *Pseudachorutes orghidani* Massoud and Gruia 1969, but differs in the number of teeth on the mandible (3 versus 4) and in the morphology of the apex of the maxilla (sharp versus hooked). Other similarities and differences among species from the Caribbean region are summarized in Table 1.

**Table 1.** Comparison of *Pseudachorutes nica* sp. n. with other Caribbean species (size in mm; Ant bulb = number of lobes in antennal apical bulb; Ant. IV = number of cylindrical sensilla; PAO = number of vesicles; PAO/E = size PAO/eye ratio; Md = number of mandibular teeth; Ocular = type of ocular setae 1, 2 and 3; Inner u = inner unguis teeth; LUT = lateral unguicular teeth); D = number of dental setae; TH = number of tenent hairs, ac = acuminate, cap = capitate.<br/>

**Species/ Character**	**Size**	**Ant bulb**	**Ant IV**	**PAO**	**PAO/E**	**Md**	**Ocular 1 2 3**	**Inner u**	**LUT**	**D**	**TH**
*Pseudachorutes nica* sp. n.	1.4	3	6	14–20	1.0	4	mMm	-	+	6	None
*Pseudachorutes orghidani*	1.2	3	5	17	1.1	3	???	+	+	6	None
*Pseudachorutes difficilis*	0.5	3	6	6–7	?	3	mmm	-	-	6	1 ac
*Pseudachorutes legrisi*	0.8	1	5?	10–13	1.8	3	mmm	+	-	6	1 cap
*Pseudachorutes reductus*	0.8	3	6?	8	1.2	5	mmm	+	-	3	1 ac

#### Variation.

The number of vesicles of postantennal organ varies asymetrically from 14 to 20. The number of circumgenital setae on the male genital plate varies from 10 to 13 and that of eugenital setae from 4 + 4 to 6 + 6. Some of the adult specimens have macrosetae with blunt tips.

## Supplementary Material

XML Treatment for Pseudachorutes nica
